# Association of gut microbiome signatures with iron metabolism response to roxadustat in peritoneal dialysis patients with anemia: a cross-sectional study

**DOI:** 10.3389/fphar.2026.1862547

**Published:** 2026-07-27

**Authors:** Jiangqing Fan, Xinqi Tan, Ying Liu, Zhe Liu, Jialing Dai, Lulu Wang

**Affiliations:** 1 Department of Pharmacy, Nanjing Drum Tower Hospital, Affiliated Hospital of Medical School, Nanjing University, Nanjing, China; 2 Department of Pharmacy, Tongxiang Hospital of Traditional Chinese Medicine, Jiangxing, China; 3 Department of Pharmacy, Nanjing Drum Tower Hospital, School of Basic Medicine and Clinical Pharmacy, China Pharmaceutical University, Nanjing, China; 4 Department of Nephrology, Nanjing Drum Tower Hospital, Affiliated Hospital of Medical School, Nanjing University, Nanjing, China; 5 The First Affiliated Hospital of Bengbu Medical University, Bengbu, Anhui, China; 6 School of Pharmaceutical Sciences, Nanjing Tech University, Nanjing, China; 7 Hunan Provincial Key Laboratory of the Research and Development of Novel Pharmaceutical Preparations, The “Double-First Class” Application Characteristic Discipline of Hunan Province (Pharmaceutical Science), Changsha Medical University, Changsha, China

**Keywords:** Anemia, chronic kidney disease, cross-sectional study, gut microbiota, iron metabolism, peritoneal dialysis, roxadustat

## Abstract

**Background:**

Anemia in chronic kidney disease (CKD) is associated with increased cardiovascular risk, impaired quality of life, and reduced survival. Roxadustat, a hypoxia-inducible factor prolyl hydroxylase inhibitor (HIF-PHI), has demonstrated non-inferior efficacy to erythropoiesis-stimulating agents (ESAs) for anemia correction in CKD. Gut microbiota modulate the intestinal HIF-iron metabolism axis, thereby regulating intestinal iron absorption. This cross-sectional study investigated the association between gut microbiome composition and iron-metabolism response to roxadustat, and developed a logistic regression model to identify factors associated with iron metabolism non-response in anemic patients undergoing peritoneal dialysis (PD).

**Methods:**

Demographic and clinical data were collected at study enrollment, and fecal samples underwent 16S rRNA gene sequencing. Microbial taxa associated with the iron-metabolism response to roxadustat were identified using linear discriminant analysis effect size (LEfSe), differential abundance analysis with DESeq2, and Spearman’s rank correlation analysis. Key microbial features were further selected using random forest analysis. Multivariable logistic regression models were constructed using R software (version 4.2.3). Variable selection was performed through stepwise selection based on the Akaike information criterion. Model performance was evaluated with the area under the receiver operating characteristic curve, calibration curves, and decision curve analysis. Internal validation was performed using 10-fold cross-validation and bootstrapping with 1,000 iterations.

**Results:**

The overall iron-metabolism response rate to roxadustat was 39% among the enrolled participants. Random forest analysis identified microbial features associated with the iron-metabolism response to roxadustat. The final multivariable model included Clostridium perfringens, Propionibacterium acnes, Bacilli, Paraeggerthella hongkongensis, compound α-ketoacid tablets, and antihypertensive medication. The final model achieved an AUC exceeding 0.8, with favorable calibration and clinical utility, and showed good discriminative performance for iron metabolism non-response to roxadustat in PD patients.

**Conclusion:**

Distinct gut microbiome signatures are associated with the iron metabolism response to roxadustat in anemic PD patients.

## Introduction

Anemia is a prevalent and clinically significant complication of chronic kidney disease (CKD), driven by relative erythropoietin (EPO) deficiency, impaired erythropoiesis, and dysregulated iron homeostasis (Chinese medical Physicians associati on nephrologist branch nephrology guidelines working group, 2021). CKD-associated anemia is associated with impaired quality of life, accelerated CKD progression, elevated cardiovascular risk, and increased all-cause mortality (Jodie et al., 2021). The burden of CKD-associated anemia remains disproportionately high in patients with end-stage kidney disease (ESKD) receiving peritoneal dialysis (PD), with a prevalence exceeding 50% and persistently suboptimal treatment responses representing a key unmet clinical need (Zhang et al., 2012; Kidney Disease: Improving Global Outcomes KDIGO Anemia Work Group, 2026).

Roxadustat, an orally active hypoxia-inducible factor prolyl hydroxylase inhibitor (HIF-PHI), is the first small-molecule agent approved for the treatment of anemia in CKD (National Medical Products Administration, 2025). It inhibits prolyl hydroxylase domain (PHD) enzymes, thereby stabilizing hypoxia-inducible factor α (HIF-α) subunits and activating downstream HIF-mediated transcription. In the intestinal epithelium, HIF-2α upregulates the expression of duodenal cytochrome b reductase (DcytB), divalent metal transporter 1 (DMT1), and ferroportin, thereby enhancing duodenal iron absorption and suppressing hepcidin synthesis (Schwartz et al., 2019; Kurata et al., 2020). The ALTAI Phase4 trial confirmed that roxadustat was associated with decreased hepcidin and also increased transferrin, soluble transferrin receptor, and total iron-binding capacity (TIBC) (Wu et al., 2024). Gut microbial metabolites modulate the intestinal HIF-iron axis by inducing epithelial hypoxia and inhibiting PHD activity, further stabilizing HIF-2α and maintaining systemic iron homeostasis (Yasmeen et al., 2022; Das et al., 2020; Parada Venegas et al., 2019; Singhal and Shah, 2020). Specifically, SCFAs produced by *Bacteroides* and *Clostridium* species augment HIF signaling in intestinal epithelial cells, whereas gut-derived uremic toxins, such as indoxyl sulfate, disrupt the HIF pathway and impair erythropoietin production (Shi-jin et al., 2022; Sampaio-Maia et al., 2016). Dysbiosis in CKD exacerbates anemia through excessive hepcidin secretion and iron metabolism disturbances (Nallu et al., 2017; Wang et al., 2026). Taken together, these findings suggest that the crosstalk between roxadustat and the gut microbiome may represent a potential therapeutic target, highlighting the potential value of microbiome profiling in optimizing treatment responses in CKD-associated anemia.

Routine therapeutic drug monitoring of roxadustat in ESKD patients receiving PD is frequently confounded by polypharmacy interactions and substantial inter-individual variability in treatment response, limiting its clinical utility. The gut microbiota has emerged as a key modulator of drug pharmacodynamics and treatment response variability across multiple chronic diseases (Zimmermann et al., 2019; Weersma et al., 2020). Accordingly, this cross-sectional study aimed to identify key factors associated with the iron-metabolism response to roxadustat by integrating 16S rRNA gene amplicon sequencing and clinical factor analysis, with the goal of developing a risk-stratification model to assist in the early identification of high-risk patients who may benefit from more intensive monitoring and individualized adjuvant therapy.

## Methods

### Study participants

This study was conducted in accordance with the Declaration of Helsinki and approved by the Medical Ethics Committee of Nanjing Drum Tower Hospital (approval No. 2023–118-01). Between March 30, 2023, and March 30, 2024, we enrolled patients with CKD stage 5 receiving maintenance PD and diagnosed with CKD-associated anemia at our center. Patients were eligible for inclusion if they had a diagnosis of CKD-associated anemia and CKD stage 5, were receiving maintenance PD and regular roxadustat treatment, were aged ≥ 18 years, were permanent residents of Nanjing, and were not participating in any other interventional clinical trials. Patients were excluded if they were receiving maintenance hemodialysis, were treated with blood transfusion or iron monotherapy for renal anemia, were using microbiota-modulating agents or antibiotics within 3 months prior to enrollment, had documented gastrointestinal disorders or severe cardiac, cerebral, and pulmonary comorbidities, or refused to provide written informed consent.

### Study medication

Roxadustat capsules (20 mg and 50 mg strengths, NMPA approval No. H20180024) were used in this study. Doses were titrated by attending physicians based on a comprehensive assessment of each patient’s hemoglobin level, body weight, therapeutic response, drug tolerability, and other clinical characteristics.

### Data collection

Demographic and clinical data were extracted from the Hospital Information System. Records with missing or implausible values were excluded. The extracted variables included demographic characteristics, hospitalization records, physical examination findings, clinical diagnoses, PD parameters, and roxadustat dosing details. The parameters of iron metabolism were recorded serum ferritin (SF), transferrin saturation (TSAT), transferrin (Tf), serum iron (SI), unsaturated iron-binding capacity (UIBC), hepcidin and total iron-binding capacity (TIBC).

### Assessment of iron-metabolism response to roxadustat and patient grouping

All participants were assessed at the cross-sectional time point at enrollment. The iron-metabolism response to roxadustat was defined by SF > 100 μg/L and TSAT>20% (Chinese medical Physicians associati on nephrologist branch nephrology guidelines working group, 2021), with patients stratified into responder (RFeY) and non-responder (RFeN) groups. Given the cross-sectional design, this classification reflects the iron-metabolism status at the time of assessment.

### 16S rRNA amplicon sequencing of gut microbiota

Fresh fecal samples (approximately 20–30 g) were collected from the mid-portion of each patient’s stool specimen and immediately stored at −80 °C until processing. All samples were collected at the cross-sectional time point at enrollment. The genomic DNA was extracted from fecal samples using the cetyltrimethylammonium bromide (CTAB) method, and sequencing libraries were prepared according to previously established protocol (Caporaso et al., 2012; Kozich et al., 2013). The V4 hypervariable region of the bacterial 16S rRNA gene was amplified using the 515F/806R primer pair (forward: 5′-GTGCCAGCMGCCGCGGTAA-3′; reverse: 5′-GGACTACHVGGGTWTCTAAT-3′) (Caporaso et al., 2012; Kozich et al., 2013). Paired-end sequencing was performed on the Illumina NovaSeq platform. Raw sequencing data were processed wuth the DADA2 plugin in QIIME 2, which encompasses quality control, denoising, read merging, and chimera removal, ultimately generating amplicon sequence variants (ASVs) (Callahan et al., 2016; Caporaso et al., 2010). Taxonomic annotation was performed by aligning representative ASV sequences against the bacterial 16S rRNA gene sequences in the Greengenes database (version. 13.8) (DeSantis et al., 2006). To ensure annotation reliability, ASVs with ambiguous taxonomic assignments or of non-bacterial origin, along with their corresponding sequences, were excluded from further analysis. Based on ASV abundance and taxonomic annotation, the proportion of sequences classified at each taxonomic level was calculated for every sample. A higher proportion of annotations at the genus or species level indicates improved taxonomic resolution. Multiple analytical approaches were employed to explore taxonomic differences between groups across various taxonomic levels, including analysis of composition of microbiomes (ANCOM), Kruskal–Wallis test, analysis of variance (ANOVA), linear discriminant analysis Effect Size (LEfSe), and DESeq2 (Mandal et al., 2015; White et al., 2009). LEfSe was used to identify microbial taxa with linear discriminant analysis (LDA) scores >2 as characteristic biomarkers for each group (Segata et al., 2011). DESeq2 analysis was performed using the “DESeq2” R package to detect differentially abundant microbes between groups (Anders and Huber, 2010). Spearman’s rank correlation coefficients were computed to evaluate associations between clinical phenotypes and microbial taxa, and correlation heatmaps were generated using the pheatmap package in R (Paliy and Shankar, 2016; Xia and Sun, 2017). To investigate the relationship between iron metabolism response to roxadustat and gut microbial communities, we utilized a correlation heatmap and Spearman’s correlation coefficients to assess the associations between specific microbial taxa and treatment outcomes. Key microbial communities that significantly influenced iron metabolism response to roxadustat were identified through random forest analysis.

### Development and validation of the risk stratification model for iron metabolism response to roxadustat

Given the cross-sectional study design, logistic regression analysis was performed to identify factors associated with iron metabolism response to roxadustat. Sample size estimation was based on the 10 events per variable (EPV) rule for logistic regression modeling (Peduzzi et al., 1996). All data preprocessing and categorical variable encoding were performed using SPSS version 26.0. Categorical variables were summarized as counts and percentages. The binary outcome was iron metabolism nonresponse and response to roxadustat. Model development, visualization, and validation were implemented in R software (version 4.2.3). The dataset was imported with the “readr” package, and categorical predictors were encoded as factors using the ‘factor’ function. Univariate and multivariable logistic regression analyses were conducted via the ‘glm’ function. Variables achieving *P* < 0.1 in univariate analysis were initially entered into the multivariable model. The final model was optimized by stepwise selection based on the minimum Akaike information criterion (AIC), allowing the retention of variables that contributed to AIC reduction even if their *P* values exceeded 0.05 in the multivariable context. For model visualization, a nomogram was constructed using the “rms” package. Discriminative performance was evaluated by receiver operating characteristic (ROC) curve analysis with the “pROC” package, and the area under the curve (AUC) was computed (Hanley and McNeil, 1982). Internal validation was performed via bootstrap resampling (1,000 iterations) and 10-fold cross-validation using the “caret” package. Model calibration was assessed with the Hosmer–Lemeshow test and calibration curves generated by the “riskRegression” package. Decision curve analysis (DCA) was conducted with the “rmda” package to assess clinical utility.

### Statistical analysis

Statistical analysis were performed using SPSS 26.0 software (IBM SPSS, United States) for demographic and clinical data. Normally distributed continuous variables are presented as mean ± standard deviation (SD), while non-normally distributed variables are reported as median (interquartile range, IQR). For between-group comparisons, independent samples t-test was used for normally distributed data with equal variances, and Welch’s t-test was used for data with unequal variances. The Mann–Whitney U test was applied for non-normally distributed data. Categorical variables are presented as frequencies and percentages and compared using the chi-square test. Graphs were generated using GraphPad Prism 9.0.0 (San Diego, United States). A two-sided *P* < 0.05 was considered statistically significant.

## Results

### Study participants

**FIGURE 1:**
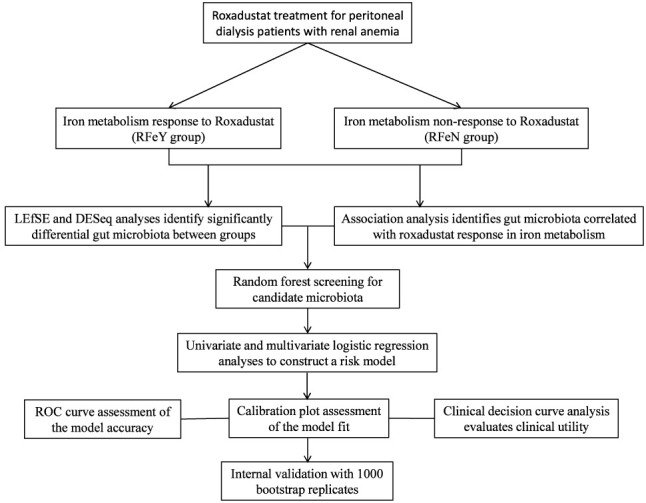
Flowchart of the study for identifying clinical and microbial factors associated with iron-metabolism response to roxadustat.

A total of 77 patients with CKD-associated anemia who were undergoing maintenance PD and treated with roxadustat were enrolled. The RFeY group comprised 30 patients, and the RFeN group comprised 47 patients. Demographic and clinical characteristics of the participants are summarized in Table 1. The study flowchart is presented in Figure 1.

**TABLE 1:** Demographic and clinical characteristics of study participants.

Variable	RFeY group	RFeN group
Total number (cases)	30	47
Age (years)	51.37 ± 2.217	55.77 ± 2.392
Male/cases (%)	18 (60%)	26 (55.3%)
BMI(kg/m^2^)	22.36 (20.445, 26.47)	22.48 (21.025, 25, 885)
Duration of peritoneal dialysis	13 (3.75, 29)	2 (0, 17)
Systolic blood pressure (mmHg)	137.5 (120.75, 151.5)	132 (122, 152)
Diastolic blood pressure (mmHg)	90.2 ± 2.143	88.53 ± 2.166
Peritoneal transport function (cases)		
Low transport	11 (68.8%)	12 (48%)
High transport	5 (31.3%)	13 (52)
Dose (mg)	70 (50,100)	70 (50,100)
Duration of treatment (months)	12 (2.5, 24)	8 (2, 17)
Underlying kidney disease/cases (%)		
Diabetic nephropathy	4 (13.8%)	11 (23.4%)
Hypertensive nephropathy	8 (27.6%)	9 (19.1%)
Primary glomerulonephritis	10 (34.5%)	11 (23.4%)
Others	7 (24.1%)	16 (34%)
eGFR (mL/min/1.73m^2^)	5.75 (4.4, 12.175)	6.5 (4.8, 9.9)

### Differences in gut microbiota composition between the RFeY and RFeN groups

**FIGURE 2:**
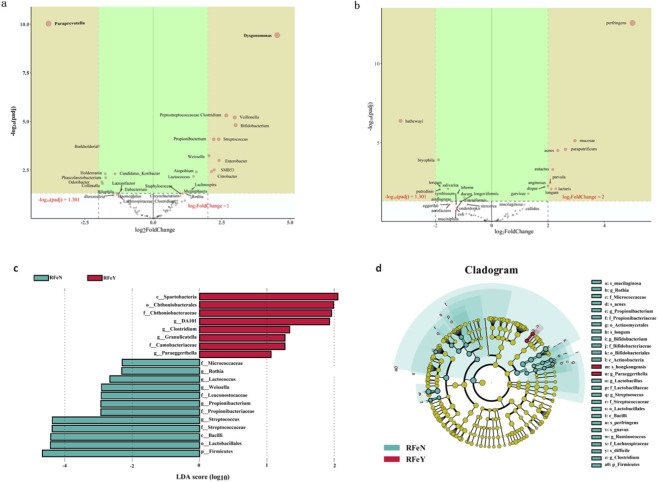
Compositional analysis of gut microbiota by DESeq2 and LEfSe. **(a)** Genus-level volcano plot of differential abundance between the RFeN and RFeY groups (DESeq2). **(b)** Species-level volcano plot of differential abundance between the RFeN and RFeY groups (DESeq2). **(c)** LDA scores of discriminative genera between the RFeY and RFeN groups (LEfSe). **(d)** Cladogram illustrating the phylogenetic distribution of species-level biomarkers discriminating the RFeY and RFeN groups (LEfSe). In the volcano plots, the x-axis shows log_2_ fold change in relative abundance, where positive values denote enrichment in the RFeN group and negative values denote depletion. A log_2_FoldChange corresponds to fourfold change. The y-axis displays statistical significance as −log_10_ (adjusted *P*), with a value of 1.301 corresponding to the adjusted *P* = 0.05 significance threshold. In the LEfSe analysis, bar lengths indicates the LDA scores, and colors indicate the group in which each taxon is enriched.

Differential abundance analysis performed with DESeq2 revealed significant differences in gut microbiota composition between the RFeY and RFeN groups. At the genus level, the RFeN group had significantly lower relative abundances of *Paraprevotella* (log_2_FC = −3.82, adjusted *P* = 9.44 × 10^−11^) and *Burkholderia* (log_2_FC = −2.00, adjusted *P* = 0.00018), and significantly higher relative abundances of *Dysgonomonas* (log_2_FC = 4.52, adjusted *P* = 3.71 × 10^−10^), *Bifidobacterium* (log_2_FC = 3.00, adjusted *P* = 1.58 × 10^−5^), and *Veillonella* (log_2_FC = 2.97, adjusted *P* = 6.28 × 10^−6^) (Figure 2a). At the species level, the RFeN group showed enrichment of *Clostridium perfringens* (log_2_FC = 4.99, adjusted *P* = 2.70 × 10^−13^), *Lactobacillus mucosae* (log_2_FC = 2.95, adjusted *P* = 7.42 × 10^−6^), and *Clostridium paraputrificum* (log_2_FC = 2.62, adjusted *P* = 2.64 × 10^−5^), and depletion of *Clostridium hathewayi* (log_2_FC = -3.23, adjusted *P* = 4.22 × 10^−7^) (Figure 2b). LEfSe analysis identified taxa with LDA scores > 2 as discriminative biomarkers. At the genus level, *Paraeggerthella*, *Granulicatella*, and *Clostridium* were enriched in the RFeY group, whereas *Rothia*, *Lactococcus*, and *Weissella* were enriched in the RFeN group (Figure 2c). At the species level, *Paraeggerthella hongkongensis* was predominant in the RFeY group, while *Clostridium difficile*, *Rothia mucilaginosa*, and *C. perfringens* were overrepresented in the RFeN group (Figure 2d).

### Correlation analysis between gut microbiota and iron metabolic response to roxadustat

**FIGURE 3:**
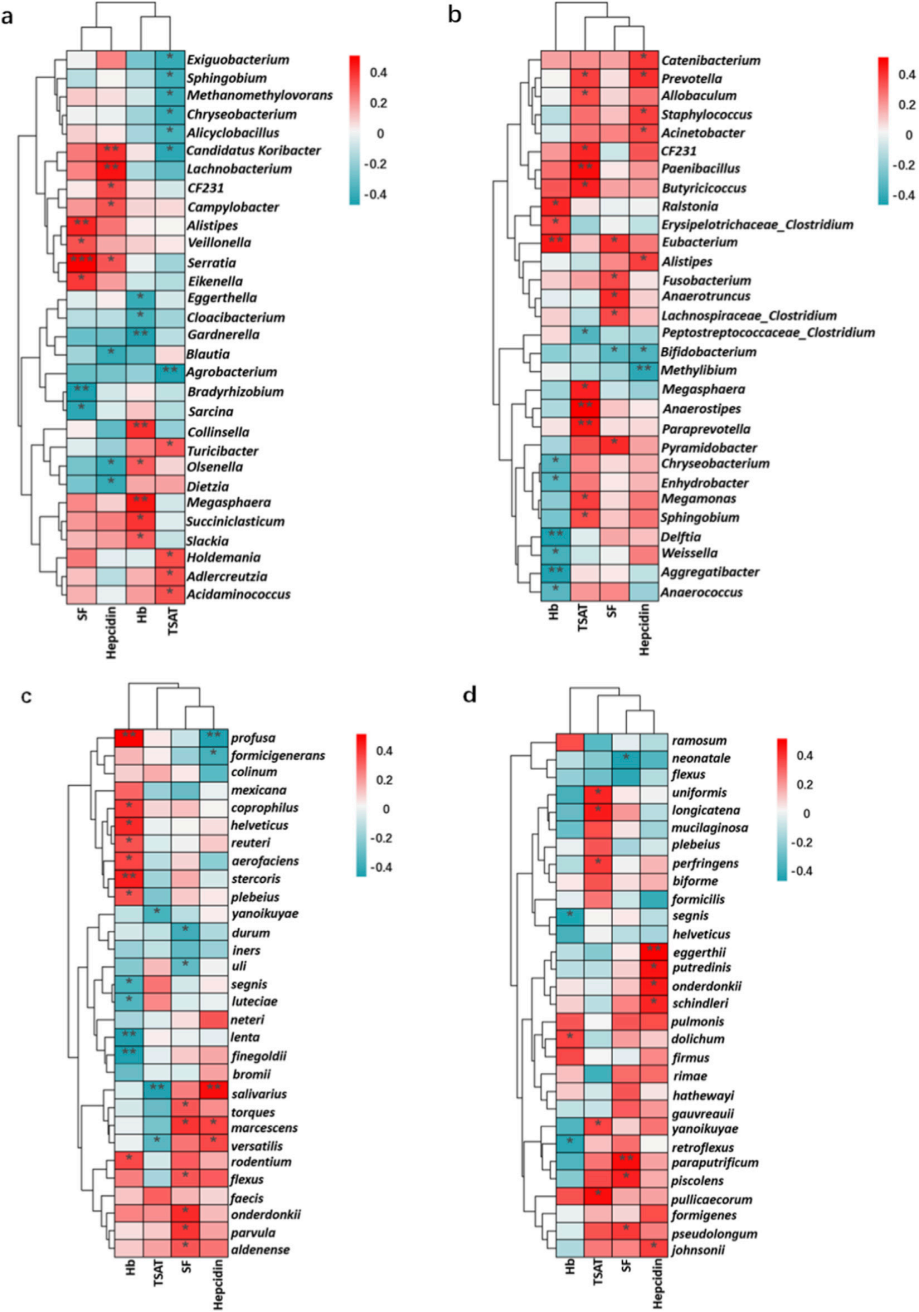
Correlation heatmaps of gut microbial taxa and indicators of the iron-metabolism response to roxadustat. **(a)** Genus-level correlation heatmap for the RFeN group. **(b)** Genus-level correlation heatmap for the RFeY group. **(c)** Species-level correlation heatmap for the RFeN group. **(d)** Species-level correlation heatmap for the RFeY group. Color intensity reflects the Spearman correlation coefficient, and asterisks denote statistical significance (*P < 0.05, **P < 0.01).

Spearman correlation analysis was performed to explore the associations between gut microbiota taxa and indicators of iron-metabolism response to roxadustat, with results visualized as correlation heatmaps (Figure 3). In the RFeN group at the genus level, SF was positively associated with *Serratia* (r = 0.4704, *P* = 0.0008) and *Alistipes* (r = 0.3986, *P* = 0.0055), and negatively associated with *Bradyrhizobium* (r = −0.4045, *P* = 0.0047). TSAT were significantly correlated with *Agrobacterium* (r = −0.4034, *P* = 0.0049),*Candidatus Koribacter* (r = −0.3687, *P* = 0.0107),and *Exiguobacterium* (r = −0.3494, *P* = 0.0160). Hepcidin were positively correlated with *Lachnobacterium* (r = 0.4303, *P* = 0.0031) and *Candidatus Koribacter* (r = 0.3805, *P* = 0.0099), and negatively correlated with *Dietzia* (r = −0.3599, *P* = 0.0151) (Figure 3a). Correlation patterns for the RFeY group at the genus level and for both groups at the species level are shown in Figures 3b–d.

### Identification of key microbiota features by random forest analysis

**FIGURE 4:**
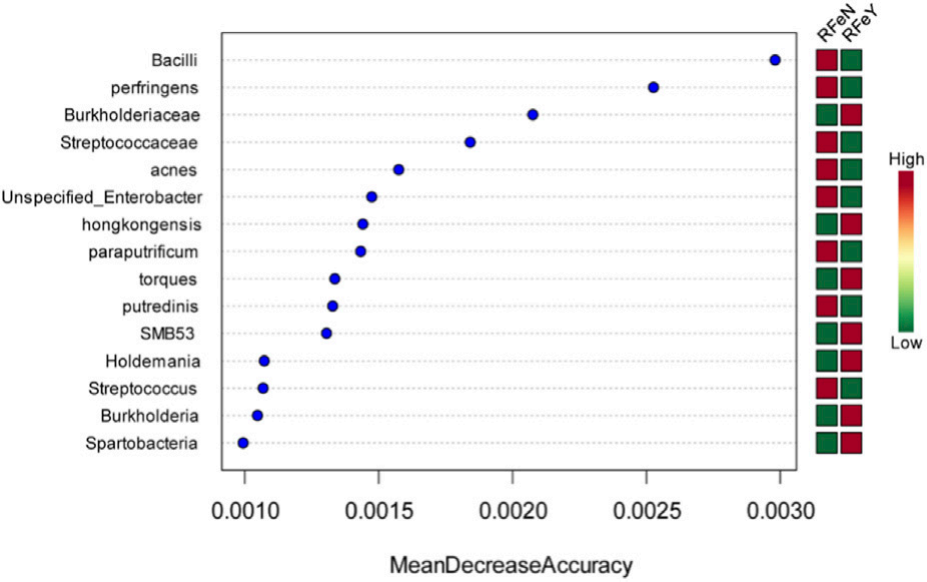
Key microbial features associated with the iron-metabolism response to roxadustat. The horizontal axis represents the feature importance score, which reflects each feature’s contribution to the discriminative performance of the random forest model. The vertical axis lists the microbial features.

Random forest analysis was applied to identify key microbial features associated with the iron-metabolism response to roxadustat, based on the differentially abundant and significantly correlated taxa previously screened across all taxonomic levels. Seven microbial features were identified as the most influential for discriminating between the RFeY and RFeN groups: *Bacilli*, *Clostridium perfringens*, Burkholderiaceae, *Paraeggerthella hongkongensis*, *Propionibacterium acnes*, Streptococcaceae, and *Enterobacter* (Figure 4).

### Univariate and multivariate logistic regression analysis of factors associated with the iron-metabolism response to roxadustat

Univariate logistic regression analysis was first conducted to screen for factors associated with iron-metabolism non-response to roxadustat. Variables with *P* < 0.1 in univariate analysis were entered into the multivariate logistic regression model. Variable selection was performed based on the minimum AIC. The final multivariable model identified seven independent factors associated with iron-metabolism nonresponse to roxadustat: *Bacilli*, *Clostridium perfringens*, *Peptostreptococcaceae* *Clostridium*, *Propionibacterium acnes*, *Paraeggerthella hongkongensis*, compound α-ketoacid tablets, and antihypertensive medicaions (AIC = 82.96) (Table 2). Complete results of univariate analysis are presented in Supplementary Table S1.

**TABLE 2:** Univariate and multivariable logistic regression analysis of factors associated with iron-metabolism nonresponse to Roxadustat.

Variable	Univariate analysis			Multivariate analysis		
associated factors	OR	95%CI	*P*	OR	95%CI	*P*
*Bacilli* (vs.≥0.013)	0.24	0.09–0.65	0.005	0.26	0.08–0.89	0.032
*Clostridium perfringens* (vs.≥no)	0.27	0.1–0.71	0.008	0.30	0.08–1.11	0.072
*Peptostreptococcaceae* *Clostridium* (vs. no)	0.27	0.08–0.91	0.035	0.30	0.07–1.24	0.096
*Propionibacterium acnes* (vs.≥no)	0.25	0.09–0.68	0.007	0.31	0.08–1.14	0.077
*Enterobacter* (vs.≥1.06E-04)	0.39	0.15–1.01	0.052	​	​	​
*Paraeggerthella hongkongensis* (vs.≥no)	5.62	1.05–30.05	0.043	23.08	2.37–225.13	0.007
Compound α-ketoacid tablets (vs. no compound α-ketoacid tablets)	0.38	0.14–0.99	0.048	0.34	0.09–1.24	0.101
antihypertensive medications (vs. no antihypertensive medications)	0.27	0.06–1.19	0.084	0.17	0.01–2.2	0.176
Diabetes (vs. with diabetes)	0.34	0.12–0.98	0.046	​	​	​

### Risk stratification model visualization

**FIGURE 5:**
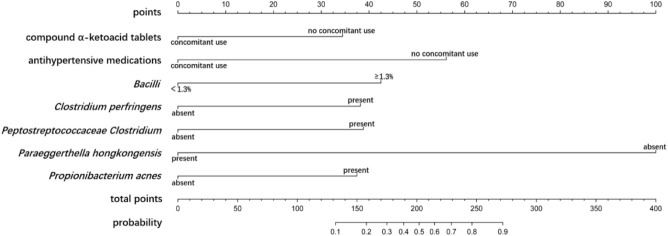
Nomograms for estimating the probability of iron metabolism nonresponse to roxadustat.

A nomogram was developed to visualize the risk stratification model based on logistic regression for iron metabolism nonresponse to roxadustat (Figure 5). To illustrate its clinical utility, we applied the nomogram to a patient profile: a 60-year-old patient receiving compound α-ketoacid tablets and not on antihypertensives. Incorporating these clinical data with the relative abundances of key microbial features (e.g., *Bacilli*: 0.01, *Clostridium perfringens*: 3.4 × 10^−5^, *Peptostreptococcaceae* *Clostridium*: 0, *Paraeggerthella hongkongensis*: 0, *Propionibacterium acnes*: 0), the nomogram yielded a total point score of 230, corresponding to an estimated 72% probability of iron-metabolism nonresponse to roxadustat.

### Risk stratification model evaluation

**FIGURE 6:**
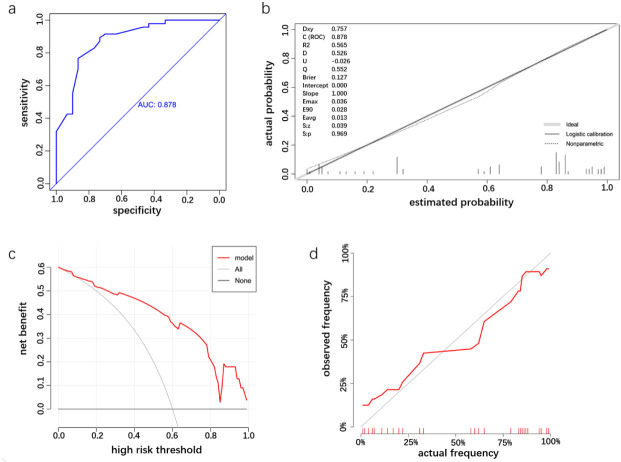
Validation of the risk stratification model for iron-metabolism nonresponse to roxadustat. **(a)** ROC curve, **(b)** calibration curve, **(c)** DCA curve, **(d)** bootstrap calibration curve.

The discriminative performance of the model was evaluated using ROC curve analysis. The AUC of the model for iron metabolism nonresponse to roxadustat was 0.878. (Figure 6a). After 1000 bootstrap resamples and 10-fold cross-validation, the AUC stabilized at 0.875, indicating good discriminative ability to distinguish between iron-metabolism responders and non-responders. Calibration was assessed using calibration curves and the Hosmer–Lemeshow test (*P* = 0.49), which demonstrated good agreement between estimated and observed probabilities of iron-metabolism nonresponse. Bootstrap calibration curves derived from 1,000 iterations further confirmed the reliability of the models (Figures 6b,d). DCA curve revealed that model yielded greater net clinical benefit than both the “treat-all” and “treat-none” strategies across a wide range of threshold probabilities (Figure 6c).

## Discussion

In this cross-sectional study, we identified key factors associated with iron-metabolism response to Roxadustat in patients with CKD-associated anemia and developed a novel risk stratification nomogram integrating demographic characteristics, comorbidities, concomitant medications, and gut microbial features. This nomogram may serve as a supportive tool to identify patients who may benefit from adjunctive iron supplementation or alternative therapeutic strategies to optimize iron metabolism.

The risk stratification model for iron-metabolism nonresponse to roxadustat incorporates multiple factors, including *Bacilli*, *Clostridium perfringens*, *Peptostreptococcaceae* *Clostridium*, *Paraeggerthella hongkongensis*, *Propionibacterium acnes,* compound α-ketoacid tablets, and antihypertensive medications, underscoring the complex interplay between gut microbiota and systemic iron homeostasis. *Bacilli* is a high-level taxon comprising diverse commensal and pathogenic bacteria with established roles in intestinal metabolic regulation and infectious disease pathogenesis (Na, 2012). *Bacilli* encompasses multiple lactic acid-producing lineages, most notably *Lactobacillus* spp., whose elevated abundance is correlated with enhanced intestinal HIF-2α signaling (Shao et al., 2022). Zhao et al. demonstrated that 3 months of roxadustat treatment increased the abundance of SCFA-producing gut microbiota and serum SCFA concentrations, accompanied by declines in circulating hepcidin and ferritin (Zhao et al., 2023). Current evidence supports the hypothesis that *Bacilli* may modulate HIF-2α signaling and iron metabolism via SCFA metabolites. Given the marked functional heterogeneity across genera within this broad taxonomic rank, the precise underlying molecular mechanisms remain to be clarified in targeted studies. *Clostridium perfringens* is an enterotoxin-producing opportunistic pathogen that contributes to various intestinal disorders (Kiu and Hall, 2018). Research on its iron-related traits is largely confined to its intrinsic iron acquisition machinery. The *C. perfringens* genome encodes the Cht locus with NEAT domain proteins ChtD and ChtE that mediate heme iron scavenging from host hemoglobin (Choo et al., 2016). As an obligate anaerobe, *C. perfringens* thrives under hypoxic conditions, and its secreted toxins further exacerbate local tissue hypoxia, which may promote host HIF-2α stabilization. The delineating its interplay with the host HIF–iron metabolism axis remains limited, warranting further mechanistic investigation. *Peptostreptococcaceae* *Clostridium* influence gut microbial community dynamics and are implicated in digestive system diseases (Si et al., 2021; Kurilshikov et al., 2021). A significant positive association was observed between the relative abundance of these taxa relative abundance and serum ferritin levels (Nonejuie et al., 2024). *Propionibacterium acnes*, a commensal skin bacterium implicated in acne vulgaris and invasive infections, produces SCFAs including propionate, under anaerobic conditions (McDowell et al., 2013; Brzuszkiewicz et al., 2011; Sanford et al., 2019). Propionate has been shown to stabilize HIF-2α via a β-oxidation-like pathway that induces cellular hypoxia (Ma et al., 2022). Given the primary skin tropism of *P. acnes* rather than intestinal colonization, its actual abundance and functional relevance in the gut ecosystem remain uncertain, which warrants further targeted validation. *Paraeggerthella hongkongensis* is an opportunistic gastrointestinal pathogen implicated in intra-abdominal infections and bacteremia (Lee et al., 2012). The scarcity of published evidence for its associations with the HIF pathway, iron metabolism, and roxadustat response warrants targeted mechanistic studies to clarify its functional roles and underlying mechanisms. The inclusion of these microbial features in our model underscores the potential influence of gut microbiota on iron metabolism status during roxadustat treatment in patients with CKD-associated anemia. Future studies are needed to clarify the effects of these microbial communities on iron metabolism response to roxadustat and their specific roles in the gut ecosystem of patients with CKD.

Regarding clinical factors, our analysis revealed that patients not receiving compound α-ketoacid tablets had a 66% higher risk of failing to meet iron metabolism targets, while those not receiving antihypertensive medications had an 83% increased risk. All patients receiving compound α-ketoacid tablets in this study were administered standard oral doses of 7–15 g per day, divided into three doses taken after meals. Compound α-ketoacid tablets are an important adjuvant therapy for low-protein dietary management in patients with CKD, consisting of four ketoamino acid calcium salts, one hydroxyamino acid calcium salt, and five amino acids. Compound α-ketoacid tablets are used to prevent and treat damage caused by protein metabolism disorders in CKD, create a more favorable internal environment for roxadustat treatment, and enhance patient response to the drug (Wang et al., 2019). Recent studies have found that in addition to improving nutritional status and correcting metabolic acidosis, α-ketoacids exert renal protective effects by modulating the gut microbiota. α-ketoacid significantly repaired intestinal barrier injury, increased the abundance of SCFA-producing genera, and decreased the abundance of *Anaerovorax* and *Coprococcus* (Mo et al., 2021). Furthermore, α-ketoacid supplementation significantly reduced serum levels of indoxyl sulfate (IS), and Spearman correlation analysis showed that the abundance of *Coprococcus* was positively correlated with serum IS levels (Mo et al., 2021). The increase in *Akkermansia* is particularly noteworthy, as this genus can increase goblet cell numbers, restore inner mucus layer thickness, and upregulate the expression of intestinal tight junction proteins (Mo et al., 2021). These findings suggest that α-ketoacids may reduce the entry of uremic toxins into the circulation by reshaping the gut microbiota and repairing the intestinal barrier, thereby creating a more favorable internal environment for roxadustat treatment. α-ketoacids may delay CKD progression by modulating the gut microbiota mediated gut–kidney axis (Zhu and Yao, 2019; Zhu et al., 2022). Compound α-ketoacids can improve microinflammatory status through multiple pathways, including improving nutritional status, correcting acid-base imbalance, regulating oxidative stress, modulating gut microbiota, and ameliorating dyslipidemia in patients with CKD (Chen et al., 2024). Existing evidence is limited to the modulatory effects of α-ketoacids on gut microbial structure, and how they interact with the HIF–iron metabolism pathway of roxadustat remains an important research gap.

With respect to antihypertensive medications, effective blood pressure control mitigates cardiovascular risk and improves cardiovascular health (Townsend and Taler, 2015). Optimal blood pressure regulation also reduces renal burden, decreases urinary protein excretion, and improves renal perfusion, which may indirectly enhance the therapeutic effects of roxadustat (Townsend and Taler, 2015; Burnier and Damianaki, 2023). These findings emphasize the potential importance of rational pharmacotherapy in improving iron metabolism and enhancing the treatment response to roxadustat in CKD patients. There is a complex bidirectional interaction between antihypertensive drugs and gut microbiota. Gut microbiota modulate the pharmacokinetics of antihypertensive drugs, whereas antihypertensive medicines alter the composition and function of the gut microbiota (Chen et al., 2022; González-Correa et al., 2024; Yang et al., 2025). The compound α-ketoacid tablets and antihypertensive agents have been well documented to modulate the composition and functional profiles of the gut microbiota. There remains a paucity of evidence regarding gut microbiota-mediated interactions between roxadustat and these concomitant medications. Roxadustat has been demonstrated to modulate the gut microbiota and reduce circulating levels of pro-inflammatory cytokines and hepcidin in patients with chronic kidney disease (Zhao et al., 2023). Theoretically, the microbiota-regulating effects of compound α-ketoacid tablets and antihypertensive agents may exert synergistic or antagonistic effects on the therapeutic efficacy of roxadustat. This hypothesis requires validation via dedicated investigations focusing on drug–microbiota–efficacy crosstalk.

Several limitations of this study should be acknowledged. Despite dietary intake being a well-recognized determinant of gut microbiota composition, detailed dietary records were not collected. This limitation is partially mitigated by the standardized peritoneal dialysis dietary counseling provided to all participants at our single center, combined with the recruitment of long-term local residents, which likely reduced inter-individual dietary heterogeneity. Dietary interventions have been shown to influence disease progression by maintaining gut microbial homeostasis and regulating metabolite levels (Chang et al., 2025). Future studies incorporating validated dietary assessment tools and controlled dietary interventions are warranted to clarify the relative contributions of diet and microbiota to iron-metabolism response to roxadustat and renal anemia outcomes. Additionally, the lack of systematic collection of participants’ long-term antibacterial use histories prior to enrollment constitutes another limitation of this study. Although we excluded patients with antibiotic or microbiota-modulating agent use within the preceding 3 months to minimize recent exposure effects, we cannot completely rule out residual confounding from earlier antibiotic use. Furthermore, given the cross-sectional design, fecal samples and relevant clinical data were collected at a single time point at enrollment. Consequently, the model was developed to screen for patients at high risk of concurrent iron-metabolism non-response to roxadustat rather than to predict future outcomes. The current findings are derived from a single-center cohort and lack external validation. Future multicenter studies with external validation, incorporating baseline predictors and longitudinal follow-up, are warranted to assess the model’s utility for prospectively identifying patients at risk of iron-metabolism non-response over time. In addition, fecal SCFA concentrations showed no significant inter-group differences and were therefore excluded from the final model. Notwithstanding these limitations, our model demonstrates robust and reliable performance. The integration of clinical parameters and gut microbiota profiling enhances its discriminative capacity, and variable selection based on the lowest AIC ensures optimal overall fit, even for variables with P-values above the conventional 0.05 threshold. Internal validation via 1,000 bootstrap resamplings confirmed good calibration and discriminative performance, and decision curve analysis indicated net clinical benefit across a range of threshold probabilities, suggesting potential value for clinical decision-making.

In conclusion, this study identifies specific gut microbial signatures associated with iron-metabolism response to roxadustat in anemic PD patients and presents an integrative model that combines clinical and microbial factors. These findings offer a foundation for personalized therapeutic stratification, though prospective multicenter studies are needed to confirm the model’s broader applicability and to elucidate the mechanistic roles of the identified microbial taxa in CKD-associated anemia pathophysiology.

## Data Availability

The raw clinical data generated and analyzed in this study are not publicly available due to ethical restrictions and patient privacy protection requirements, as the data involve sensitive personal health information of enrolled human participants. De-identified data may be available from the corresponding author upon reasonable written request, pending formal approval from the institutional ethics committee.
