# Supplementary material for: Association of gut microbiome signatures with iron metabolism response to roxadustat in peritoneal dialysis patients with anemia: a cross-sectional study
Source: Front Pharmacol. 2026 Jul 27;17:1862547. doi: 10.3389/fphar.2026.1862547 (PMC13454119; doi:10.3389/fphar.2026.1862547)
Supplement: Supplementary file 1 [file Table1.docx]

**Supplement data**

**Table S1 Univariate and multivariate logistic regression analysis of iron metabolism non-response to roxadustat in renal anemia patients**

| Variable | Univariate analysis | | | Multivariate analysis | | |
| --- | --- | --- | --- | --- | --- | --- |
| **Associated microbiota** | OR | 95%CI | *P* | OR | 95%CI | *P* |
| *Acinetobacter johnsonii*(vs.≥2.06E-04) | 0.54 | 0.21-1.36 | 0.192 |  |  |  |
| *Moraxellaceae*(vs.≥7.9E-04) | 0.62 | 0.25-1.55 | 0.306 |  |  |  |
| *Parvimonas*(vs.≥no) | 0.81 | 0.31-2.1 | 0.659 |  |  |  |
| *Flavobacteriia* (vs.≥6.68E-05) | 0.77 | 0.31-1.93 | 0.577 |  |  |  |
| *Acinetobacter* (vs.4.78E-04) | 0.77 | 0.31-1.93 | 0.577 |  |  |  |
| *Aggregatibacter segnis* (vs. no) | 1.29 | 0.32-5.26 | 0.720 |  |  |  |
| *Chryseobacterium* (vs.≥6.28E-05) | 0.62 | 0.25-1.55 | 0.306 |  |  |  |
| *Enhydrobacter* (vs.≥2.76E-04) | 0.49 | 0.19-1.25 | 0.138 |  |  |  |
| *Sphingobium Yanoikuyae* (vs.≥1.25E-04) | 0.77 | 0.31-1.93 | 0.577 |  |  |  |
| *Weeksellaceae* (vs.≥6.68E-05) | 0.77 | 0.31-1.93 | 0.577 |  |  |  |
| *Burkholderia* (vs.≥2.19E-05) | 0.62 | 0.25-1.55 | 0.306 |  |  |  |
| *Bacilli* (vs.≥0.013) | 0.24 | 0.09-0.65 | 0.005 | 0.26 | 0.08-0.89 | 0.032 |
| *Clostridium perfringens* (vs.≥no) | 0.27 | 0.1-0.71 | 0.008 | 0.30 | 0.08-1.11 | 0.072 |
| *Burkholderiaceae* (vs.≥2.48E-05) | 0.96 | 0.38-2.39 | 0.927 |  |  |  |
| *Peptostreptococcaceae Clostridium* (vs. no) | 0.27 | 0.08-0.91 | 0.035 | 0.30 | 0.07-1.24 | 0.096 |
| *Propionibacterium acnes* (vs.≥no) | 0.25 | 0.09-0.68 | 0.007 | 0.31 | 0.08-1.14 | 0.077 |
| *Enterobacter* (vs.≥1.06E-04) | 0.39 | 0.15-1.01 | 0.052 |  |  |  |
| *Paraeggerthella hongkongensis* (vs.≥no) | 5.62 | 1.05-30.05 | 0.043 | 23.08 | 2.37-225.13 | 0.007 |
| **Roxadustat Treatment Status** |  |  |  |  |  |  |
| Roxadustat Dosage (vs. 50 mg/dose) |  |  |  |  |  |  |
| 100 mg/dose | 1.87 | 0.53-6.56 | 0.330 |  |  |  |
| 70 mg/dose | 0.77 | 0.27-2.18 | 0.622 |  |  |  |
| Roxadustat Duration (vs. ≥12 months) | 1.24 | 0.49-3.1 | 0.648 |  |  |  |
| **Concomitant Medications** |  |  |  |  |  |  |
| Antidiabetic Agents (vs. no antidiabetic agents) | 0.44 | 0.15-1.29 | 0.135 |  |  |  |
| α-Ketoglutarate (vs. no α-ketoglutarate) | 0.38 | 0.14-0.99 | 0.048 | 0.34 | 0.09-1.24 | 0.101 |
| Febuxostat (vs. no febuxostat) | 0.68 | 0.27-1.71 | 0.410 |  |  |  |
| Bailing Capsules (vs. no Bailing capsules) | 1.14 | 0.33-4 | 0.834 |  |  |  |
| Sucrose Iron Injection (vs. no sucrose iron) | 0 | 0-Inf | 0.992 |  |  |  |
| Diuretics (vs. no diuretics) | 0.67 | 0.22-2.1 | 0.497 |  |  |  |
| Sodium Bicarbonate Tablets (vs. no sodium bicarbonate) | 0.54 | 0.19-1.51 | 0.239 |  |  |  |
| Ferrous Succinate Sustained-Release Tablets (vs. with ferrous succinate) | 0.73 | 0.28-1.93 | 0.53 |  |  |  |
| Sivelestat (vs. no sivelestat) | 0.65 | 0.22-1.96 | 0.449 |  |  |  |
| Sodium Zirconium Cyclosilicate Powder (vs. no sodium zirconium) | 1.63 | 0.31-8.66 | 0.567 |  |  |  |
| Lanthanum Carbonate Chewable Tablets (vs. no lanthanum carbonate) | 1.23 | 0.49-3.13 | 0.661 |  |  |  |
| Polysaccharide Iron Complex Capsules (vs. with polysaccharide iron complex) | 0.54 | 0.13-2.23 | 0.396 |  |  |  |
| Calcitriol Soft Capsules (vs. no calcitriol) | 0.88 | 0.34-2.31 | 0.799 |  |  |  |
| ShenKang Injection (vs. no ShenKang injection) | 0.76 | 0.17-3.3 | 0.713 |  |  |  |
| Uremic Detoxification Granules (vs. no uremic detoxification) | 1.63 | 0.31-8.66 | 0.567 |  |  |  |
| Antihypertensive Medications (vs. no antihypertensive medications) | 0.27 | 0.06-1.19 | 0.084 | 0.17 | 0.01-2.2 | 0.176 |
| Atorvastatin Calcium Tablets (vs. no atorvastatin) | 0.65 | 0.18-2.34 | 0.509 |  |  |  |
| **Comorbidities** |  |  |  |  |  |  |
| Hyperuricemia (vs. with hyperuricemia) | 0.67 | 0.27-1.69 | 0.400 |  |  |  |
| Hypoproteinemia (vs. with hypoproteinemia)90 | 0.40 | 0.12-1.38 | 0.147 |  |  |  |
| Diabetes (vs. with diabetes) | 0.34 | 0.12-0.98 | 0.046 |  |  |  |
| Hyperlipidemia (vs. with hyperlipidemia) | 0.77 | 0.13-4.47 | 0.769 |  |  |  |
| Hyperphosphatemia (vs. with hyperphosphatemia) | 0.95 | 0.34-2.67 | 0.924 |  |  |  |
| Liver and Gallbladder Diseases (vs. with liver and gallbladder diseases) | 0.97 | 0.37-2.56 | 0.949 |  |  |  |
| Immune System Diseases (vs. with immune system diseases) | 1.19 | 0.25-5.75 | 0.825 |  |  |  |
| Cardiovascular and Cerebrovascular Diseases (vs. with cardiovascular and cerebrovascular diseases) | 1.28 | 0.5-3.28 | 0.608 |  |  |  |
| Hyperthyroidism (vs. with hyperthyroidism) | 0.61 | 0.22-1.69 | 0.342 |  |  |  |
| Electrolyte Disorders (vs. with electrolyte disorders) | 0.75 | 0.2-2.75 | 0.664 |  |  |  |
| Hypertension (vs. without hypertension) |  |  |  |  |  |  |
| Stage 1 | 0.50 | 0.06-4.47 | 0.535 |  |  |  |
| Stage 2 | 2.12e+07 | 0-Inf | 0.991 |  |  |  |
| Stage 3 | 0.67 | 0.11-3.94 | 0.655 |  |  |  |
| Underlying Kidney Disease (vs. other types) |  |  |  |  |  |  |
| Chronic Nephritis | 0.56 | 0.16-2.01 | 0.377 |  |  |  |
| Hypertensive Kidney Disease | 0.55 | 0.16-1.84 | 0.331 |  |  |  |
| Diabetic Kidney Disease | 1.37 | 0.33-5.72 | 0.661 |  |  |  |
| **Demographic Data** |  |  |  |  |  |  |
| Age (vs. ≥54 years) | 0.78 | 0.31-1.95 | 0.590 |  |  |  |
| Gender (vs. female) | 0.83 | 0.33-2.09 | 0.686 |  |  |  |
| Body Mass Index (BMI) (vs. ≥24) | 1.03 | 0.41-2.6 | 0.946 |  |  |  |
| Heart Rate (vs. ≥80 beats/min) | 0.85 | 0.33-2.19 | 0.742 |  |  |  |
| Systolic Blood Pressure (vs. ≥136 mmHg) | 1.30 | 0.52-3.25 | 0.577 |  |  |  |
| Diastolic Blood Pressure (vs. ≥89 mmHg) | 1.30 | 0.52-3.25 | 0.577 |  |  |  |
| Duration of Peritoneal Dialysis (vs. PD catheter placement) |  |  |  |  |  |  |
| ≤12 months | 0.68 | 0.18-2.55 | 0.571 |  |  |  |
| >12 months | 0.43 | 0.13-1.48 | 0.183 |  |  |  |
| **Subclinical inflammatory markers** |  |  |  |  |  |  |
| Albumin | 1.96 | 0.77-5.02 | 0.159 |  |  |  |
| CRP | 0.65 | 0.2-2.12 | 0.479 |  |  |  |
| Neutrophil percentage | 1.14 | 0.45-2.84 | 0.785 |  |  |  |
| Lymphocyte percentag | 0.8 | 0.32-2 | 0.631 |  |  |  |
| WBC count | 0.87 | 0.35-2.18 | 0.765 |  |  |  |
